# Influence of low direct electric currents and chlorhexidine upon human dental biofilms

**DOI:** 10.1002/cre2.34

**Published:** 2016-07-22

**Authors:** Jérôme F. Lasserre, Selena Toma, Thomas Bourgeois, Hajar El Khatmaoui, Estelle Marichal, Michel C. Brecx

**Affiliations:** ^1^ Department of Periodontology, School of Dentistry Saint‐Luc University Hospital, Université catholique de Louvain Brussels Belgium; ^2^ Institut de Recherche Expérimentale et Clinique, Pôle de Morphologie Université catholique de Louvain Brussels Belgium

**Keywords:** Bioelectric effect, chlorhexidine, dental biofilm, peri‐implantitis

## Abstract

Dental biofilms have been widely associated with biological complications of oral implants. Currently, no consensus exists regarding the most reliable anti‐infective approach to treat peri‐implantitis. This study aimed to investigate whether low direct electric currents (DC) could influence chlorhexidine (CHX) 0.2% antimicrobial efficacy against human dental biofilms. To support biofilm accumulation, discs made with machined titanium (Ti) or hydroxyapatite (HA) were used. Five volunteers wore during 24 h an intraoral thermoformed splint on which ten specimens were bonded. Biofilms were then collected and treated ex vivo. During each antimicrobial experiment (*N* = 20 replicates), two modalities of treatment (CHX/PBS = control groups and CHX/PBS+5mA = test groups) were tested (*n* = 5 discs each) and the number of viable bacteria evaluated in LogCFU/mL at baseline, 0.5, 1, 2 and 5 min. The proportion of killed bacteria was also estimated and compared statistically at each time point between control and test groups. CHX+/−5mA induced a mean viability reduction around 90–95% after 5 min of treatment whatever the surface considered (Ti/HA). A significant difference regarding the bactericidal effect was noted on Ti surfaces after 0.5, 1 and 2 min in favor of the CHX+5mA modality when compared to CHX alone (*p* < 0.05). PBS+5mA also had a certain antimicrobial effect (58%) after 5 min on Ti surfaces. This effect was significantly higher than that observed with PBS (25%) (*p* < 0.05). This study showed that low DC (5mA) can have an antibiofilm effect and are also able to enhance chlorhexidine 0.2% efficacy against human dental biofilms grown on titanium surfaces.

Dental implants have become a reliable solution to replace missing teeth using fixed osseointegrated anchorages (Moraschini et al. 2015). Nevertheless, during the last years, biological complications related to the nature and the physiology of dental biofilms have emerged (Mombelli et al. 2012). Indeed, the survival of an oral implant mainly depends on a balance between the oral microbiota and the host immune system. A breakdown between these two players will result in peri‐implant bone loss and ultimately to the implant failure through a phenomenon that has been named peri‐implantitis (Mombelli and Lang 1998). Peri‐implantitis is indeed an inflammatory process characterized by bleeding on probing and radiographic bone loss around the implant (Lindhe and Meyle 2008). Although recent findings and consensus meetings underlined that peri‐implant diseases display some particularities regarding their physiopathology (Becker et al. 2014; Lang and Berglundh 2011), their histopathology (Carcuac and Berglundh 2014), and their microbiomes (Kumar et al. 2012), these biofilm‐induced inflammatory diseases are often and in many ways compared with periodontitis that is related to a switch from a symbiotic to a dysbiotic microbiota (Hajishengallis and Lamont 2012).

The main objective of peri‐implantitis treatment is thus anti‐infective. It aims at disorganizing the implant‐related biofilm and to clean as well as possible the contaminated surfaces in order to recover a biocompatible implant and to reduce inflammation that causes disease progression.

Various treatment protocols have been proposed in the literature to treat peri‐implant diseases, but currently, no consensus has emerged to recommend one method over another (Esposito et al. 2012). Actually, as the etiology and physiopathology of periodontitis and peri‐implantitis are quite similar, most of peri‐implantitis therapeutic protocols are derived from periodontal treatments (Renvert et al. 2008). Nonsurgical procedures with curettes have been evaluated clinically and failed to demonstrate significant improvement (Renvert et al. 2008, 2009).

A surgical approach seems to be more effective because of better results regarding the improvement of clinical parameters, but it appears that complex and expensive protocols are not proven to be more valuable than simple mechanical debridement (Esposito et al. 2012). Furthermore, if the primary outcome of peri‐implantitis treatment is to control completely peri‐implant inflammation, very few evidence demonstrate that peri‐implantitis can be reliably treated (Toma et al. 2014).

Two reasons can be proposed to explain the difficulty to stabilize peri‐implant diseases. First, the implant surface is really challenging to decontaminate mechanically because of its threads and microroughness that have been developed initially to improve osseointegration and reduce early failures (Sahrmann et al. 2013). Secondly, microorganisms that can access the implant surface will develop as dental biofilm communities in which they are particularly resistant to antimicrobial agents and to the host immune system (Costerton et al. 1999).

If the treatment of implant surfaces with chlorhexidine (CHX), for instance, did not cause damage on different types of implant surfaces, it was not able by itself to remove already existing biofilm from such surfaces (Augthun et al. 1998). Hence, new antimicrobial therapeutic approaches would be of interest against dental biofilms to improve clinical outcomes of peri‐implantitis treatment.

One interesting approach would be to use electric currents (DC). Indeed, implants are made of titanium (Ti), a metal that could allow through its conductivity an electrochemical disinfection (Mohn et al. 2011). This purification method is already known for water decontamination and based on the creation of active substances on the electrodes. In the presence of chloride ions, oxidizer agents like chlorine are produced and play a key role for electrochemical disinfection (Jeong et al. 2009). It has been demonstrated in *in vitro* models that even alone, the use of a weak electric current could be efficient to decontaminate partially an implant surface (Mohn et al. 2011; Sahrmann et al. 2014). Moreover, an enhanced antimicrobial effect of several industrial biocides and antibiotics has been shown through the addition of low intensity direct electric currents against bacterial biofilms (Blenkinsopp et al. 1992; Costerton et al. 1994). This namely bioelectric effect, combining antimicrobials and electric currents, could also be interesting to develop new approaches for taking care of peri‐implant diseases.

Up to now, this phenomenon has only been tested in the dental research field with *in vitro* mono‐ or dual‐species biofilms grown on hydroxyapatite (HA) discs (Lasserre et al. 2015; Wattanakaroon and Stewart 2000). It would thus be relevant to study this effect on multispecies human dental biofilms to validate the relevance of this concept for further possible clinical investigations.

The aim of the present study was to test *ex vivo* the influence of 5 mA direct electric currents on the antimicrobial efficacy of CHX against human dental biofilms grown *in vivo* on titanium or HA surfaces.

## Material and Methods

### Samples

According to each experiment, two different materials/surfaces were used to support biofilm formation.

1. Grade 5 (TA6V4) machined Ti discs (Southern Implants®, Irene, South Africa) (5‐mm diameter/2‐mm width) or
2. Ceramic HA discs of the same dimensions (Clarkson Chromatography Products Inc., South Williamsport, PA, USA)

### Surface characterization

Before biofilm formation, the discs were analyzed for their morphology and surface chemical composition using scanning electron microscopy (SEM) and energy‐dispersive X‐ray (EDX) (JEOL 7200, operating at 15 kV). The discs did not require any special preparation for the SEM observation. The samples were fixed to the stubs and placed into the vacuum chamber, and the central parts of the discs were imaged at magnifications ×25 and ×500. For EDX, the ZAF method standardless quantitative analysis was used, and each chemical element found at the observed surface was evaluated in mass (mass%) and atomic (atom%) percentages.

Hydrophily of the two types of surface (Ti and HA) was also analyzed through contact angle measurements. To this end, a drop shape analysis system (DSA 10‐MK2; Kruess, Hamburg, Germany) equipped with a digital camera and image analysis was used. Ultrapure water at 25 °C (Simplicity 185 UV; Millipore, Billerica, MA) with a drop size of 3 μL was used as wetting liquid. Contact angles of the air‐water‐substrate interface were measured three times on three specimens and for each tested material. Finally, surface roughness was evaluated with a profilometer (DektakXT, Bruker, Bruker Nano Surfaces Division, Tucson, AZ, USA) as to register *R*a and *R*z values plus standard deviations for the two types of surfaces (Ti and HA) (*N* = 4 replicates for each surface). To do so, an 0.7‐µm stylus was moved in contact with the tested specimen during 60 sec, with a 1‐mg force and on 2.2 mm from the center of the slab. Data were acquired and analyzed with the Vision64® 5.40 software (Bruker Corp., USA).

### Dental biofilm formation

To support biofilm formation *in vivo*, five healthy volunteer students (three females and two males) between 22 and 37 years old were taken alginate impressions of their maxilla, and from these impressions, dental casts were obtained. None of the participants had taken systemic antibiotics nor oral antiseptics for the 6 months preceding the start of the study. None of them was smoker, and they all presented good periodontal health and excellent plaque control with full‐mouth bleeding and plaque scores close to zero. Before the experiments, all the volunteers gave their informed consent to participate to the study.

**Figure 1:**
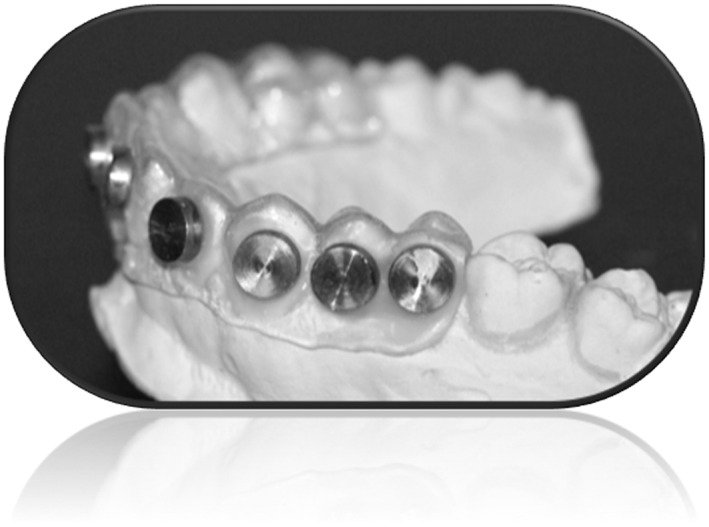
Titanium or hydroxyapatite discs were attached in vestibular position from tooth no. 15 to no. 25 (*N* = 10 specimens) on intraoral removable splints to support *in vivo* dental biofilm formation.

After production of the dental casts, intraoral removable individualized customized thermoformed polyethylene splints were produced and 10 titanium or HA discs were attached on the vestibular faces from teeth no. 15 to no. 25 (five specimen on each side = 10 specimen on every splint) using cyanoacrylate glue (Omnident®, Rodgau, Germany) (Fig. 1).

Splints with the disks were then sterilized with ethylene oxide and conserved 48 h before wearing to allow gas desorption and avoid any allergic or toxic reaction.

Dental splints were then worn in the mouth by the participants during 24 h to permit dental biofilm formation. During this period, no oral hygiene was allowed and splints had to remain in the mouth at all time except during meals for which they had to be placed in a glass of warm water. In total, the five volunteers repeated 20 experiments (*N* = 20 replicates, i.e. 200 biofilm samples). Fourteen experiments were performed with Ti samples and six with HA.

### Biofilm killing treatment

After each experiment, 10 biofilms were formed and ready to be treated individually according to one of the following procedures: CHX 0.2% (five discs) or CHX 0.2% + 5 mA DC (five discs).

**Figure 2:**
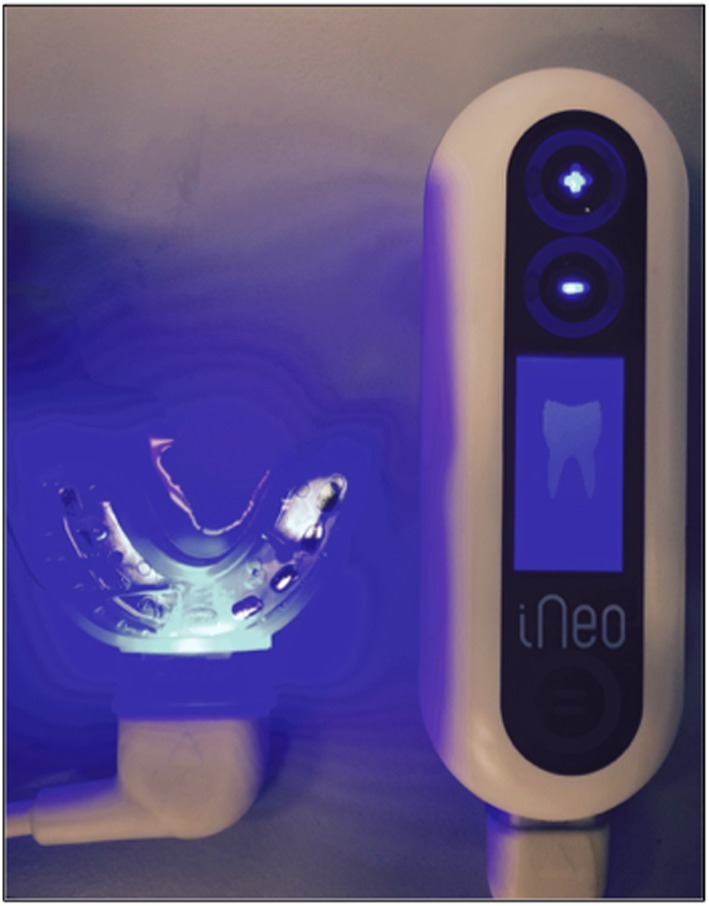
iNéo experimental device allowing the production of 5 mA DCs in the illuminated recipient during the *ex vivo* antimicrobial procedure of the human dental biofilms.

Indeed, two series of five discs/biofilms were removed aseptically from the splints with Friedman Gouge forceps after each experiment and prepared for the *ex vivo* biofilm killing assay. The first series (five samples) was only submitted, through immersion of the discs, to the action of CHX previously poured into a sterile Petri dish (Corsodyl®, GlaxoSmithKline, UK). The second one (five samples) was additionally and concomitantly treated with a 5 mA DC allowed by the activation of a customized electrified tray (Inéo™, SATELEC, Acteon Group, Mérignac, France) (Fig. 2). This experimental device only allows for 5 mA DCs so that it was not possible to test several other currents. Each modality of treatment was applied during 5 min, and its bactericidal efficacy evaluated after 30 sec, 1, 2, and 5 min. To do so, at each time point (T0, 30 sec, 1, 2, 5 min), one disc was removed from the antiseptic solution, washed during 3 sec with sterile phosphate‐buffered saline (PBS), and transferred in a microtube containing 1 mL of PBS.

### Bacterial culture and cell counts

Afterwards, the microtubes containing one disc each were vortexed during 1 min and ultrasonicated for 1 min (100 W, 42 KHz) to detach sessile bacteria as proposed by Mohn et al. (2011). Finally, these recovered bacteria were cultured as described in the succeeding texts, in order to evaluate the antibacterial effect of each treatment modality on the biofilm.

More precisely, 100 μL of the collected biofilm bacteria were inoculated after serial 10‐fold dilutions (from 10^0^ to 10^−3^) on enriched blood agar plates. They were thus cultured on Columbia agar plates (pH = 7.3) (BBL™ Columbia Agar Base, Becton, Dickinson and Company, Sparks, MD, USA) enriched with 5 mg/L of hemin and 1 mg/L of vitamin K. All plates were then transferred for incubation within 15 min into the Bugbox® anaerobic workstation (LED Techno NV, manufactured by Ruskinn Technology Limited, Leeds, UK) of which the atmosphere was composed of 80% N_2_, 10% H_2_, and 10% CO_2_ (ANAERO 10, AIR LIQUIDE Medical, Liège, Belgium). Incubation was then allowed for 10 days at 37 °C. Afterwards, the number of colony‐forming units (CFUs) was counted by the use of a computer‐assisted device (Acolyte, from Synbiosis®, Frederick, Maryland, USA) and evaluated in CFU/mL. For each time point and each modality of treatment, averages and standard deviations were calculated and estimated in LogCFU/mL. Additionally, the mean LogCFUs and percentage reductions were also calculated for each antimicrobial procedure. Data were collected at T0, 0.5, 1, 2, and 5 min and for each treatment modality. Microbiological analyzes were performed by an independent examiner blinded to the nature of the project.

### Statistical analysis

Statistical analyses were performed using GraphPad Instat 3 sofware. CFUs per disc were averaged and submitted to logarithmic transformations. The percentage of viability reduction in each group was also calculated and compared. The significance of the obtained data was assessed using Mann–Whitney tests and unpaired *t*‐tests with Welch correction when necessary. Statistical differences were considered significant when *P* < 0.05.

## Results

### Surface characterization

**Figure 3:**
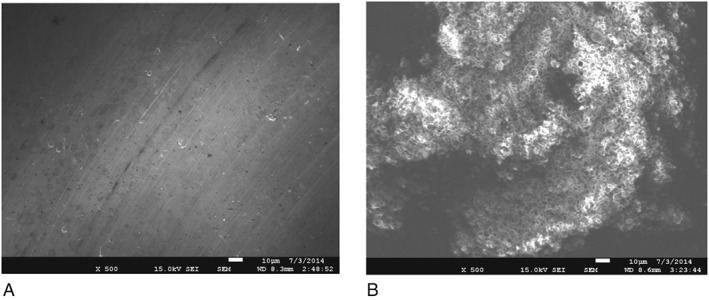
Scanning electron micrographs of titamium (A) and hydroxyapatite (B) discs used for dental biofilm formation. Notable differences can be observed with regards to their relative microtopography HA showing a much more roughened surface (×500).

**Figure 4:**
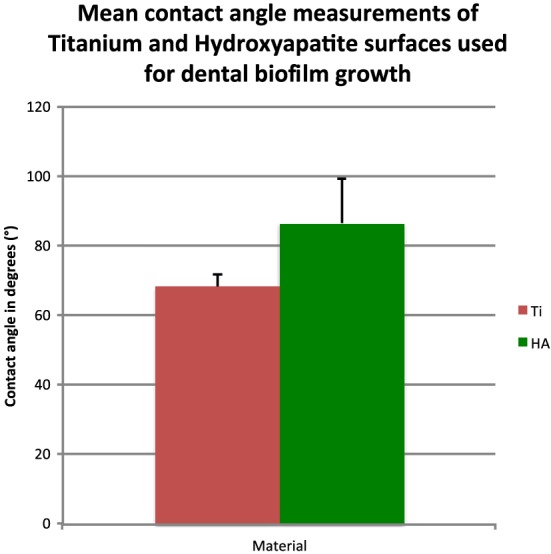
Wettability expressed in mean contact angle for water shows that both surfaces (Ti and HA) display low hydrophilicity (contact angles: 45 < θ < 90°).

Scanning electron microscopy (SEM) micrographs of Ti and HA surfaces used to support dental plaque biofilm formation are presented in Figure 3. At ×500 magnification, remarkable differences regarding surface topography can be observed: Ti machined samples present a relatively smooth surface, whereas HA samples are clearly roughened. With regards to surface wettability, contact angle measurements shown in Figure 4 indicate that both surfaces present a low hydrophilicity with mean values of 68.4° and 86.8° for Ti and HA, respectively (Fig. 4).

**Figure 5:**
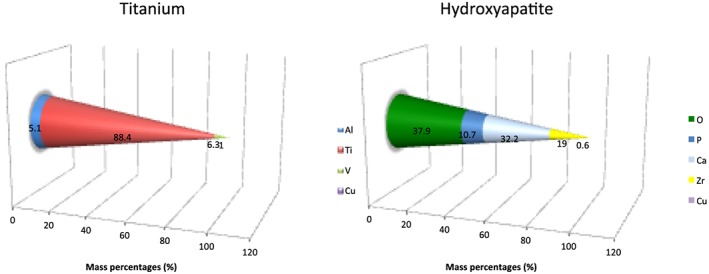
Chemical composition (mass%) of the two investigated surfaces (Ti and HA) measured by EDX elemental analysis.

Energy‐dispersive X‐ray (EDX) analysis revealed the main chemical composition of each surface samples and confirmed Ti surfaces to be grade 5 titanium containing aluminum and vanadium as additional components. The results are presented in mass% in Figure 5. Regarding roughness characteristics of the tested surfaces, the mean values ± SD for *R*a were 0.31 µm (±0.15) and 0.73 µm (±0.34) for Ti and HA, respectively. Peak‐to‐valley values (*R*z) were 1.03 µm (±0.17) for Ti and 4.62 µm (±1.66) for HA.

#### Antimicrobial efficacies of CHX 0.2% with or without 5 mA DC on dental biofilm cells grown on Ti surfaces (*N* = 9 replicates)

**Figure 6:**
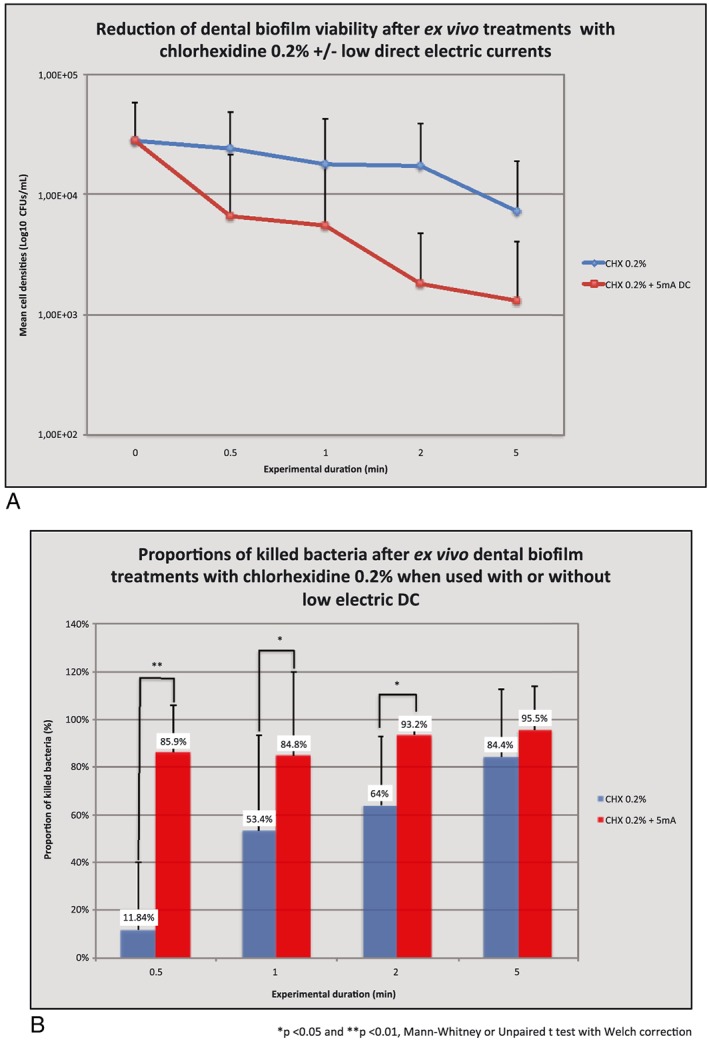
(A) Titamium specimens. (B) Titamium specimens.

The results of the action of the different antimicrobial procedures against dental biofilms are presented in mean cell densities expressed in LogCFU/mL between baseline and 5 min of treatment. Data were collected at T0, 0.5, 1, 2, and 5 min as presented in Figure 6A. For each modality and duration of treatment, proportions of killed bacteria were also calculated and compared between control (CHX) and test (CHX + 5 mA DC) groups at each time point by using Mann–Whitney or unpaired *t*‐tests with Welch correction when necessary (Fig. 6B). Data shows that both treatments are efficient in killing biofilm bacteria with respectively 84.4 and 95.5% of viability reduction for control and test groups after 5 min of antimicrobial treatment. Concerning the Ti surface, between groups comparisons showed a statistically more efficient result in viability reduction in favor of the test group (CHX + 5 mA) at 0.5, 1, and 2 min of treatment (*P* < 0.05). This was not the case after 5 min.

#### Antimicrobial efficacies of CHX 0.2% with or without 5 mA DC on dental biofilm cells grown on HA surfaces (*N* = 6 replicates)

**Figure 7:**
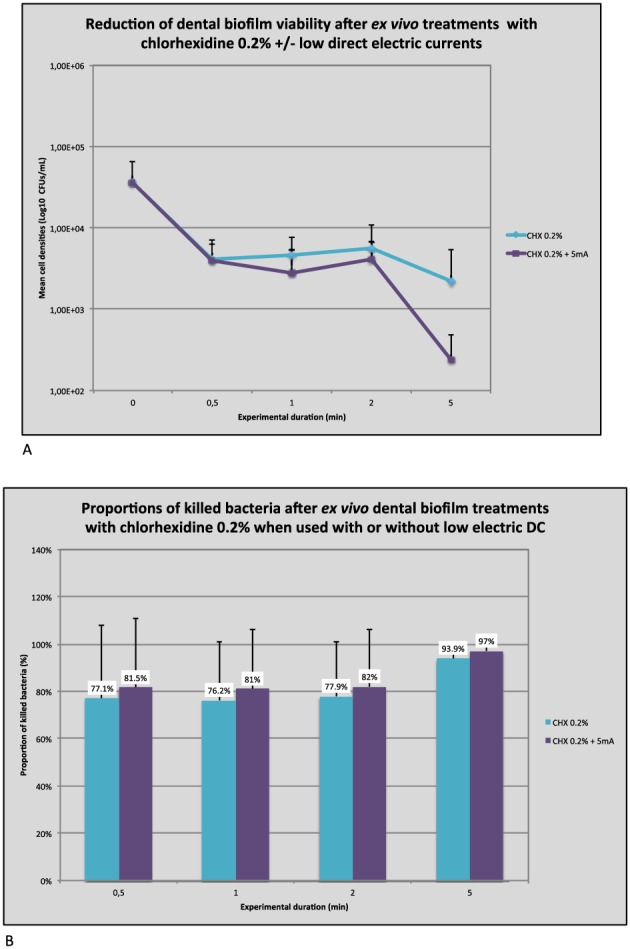
(A) Hydroxyapatite specimens. (B) Hydroxyapatite specimens.

Results regarding the antimicrobial effects of the tested modalities (CHX ± 5 mA DC) on HA are presented in Figure 7A, B. Graphs show that on HA, the viability reduction already reached 1 Log unit after 30 sec of treatment whatever the treatment used corresponding respectively to a reduction of 77.1 and 81.5% of killed bacteria for the control and the test groups. Afterwards, the viability reduction was slower, reaching after 5 min 93.9 and 97% of killed cells for control and test groups, respectively. At each time point, the between groups comparisons showed no statistical difference (*P* > 0.05).

#### Antimicrobial efficacies of PBS with or without 5 mA DC on dental plaque biofilm cells grown on Ti surfaces (*N* = 5 replicates)

**Figure 8:**
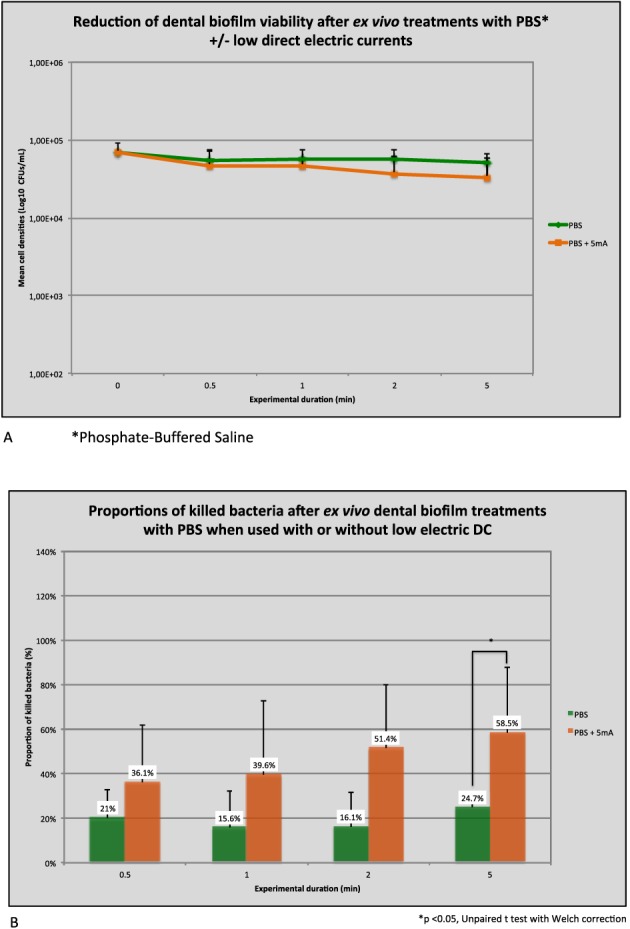
(A) Titamium specimens. (B) Titamium specimens.

In order to see whether the significant impact of electrical currents on CHX activity observed on Ti surfaces could be attributed to a synergistic effect, additional experiments were performed to compare the viability reduction of the dental biofilm submitted *ex vivo* during 5 min to PBS with or without 5 mA DCs. Log cell densities and viability reductions are presented in Figure 8A, B. The cell number density decrease was minimal using PBS reaching around 25% after 5 min. The additional use of 5 mA currents was not statistically significant in enhancing viability reduction up to 2 min. However, a slight improvement was noted in favor of this group, and this trend became significant after 5 min as shown with a Student's *t*‐test analysis (*P* = 0.03). At T5, the proportion of killed bacteria compared with baseline was more than twice as in the control group with a percentage of viability reduction increasing up to 58.5%.

## Discussion

The present research tested the hypothesis that weak direct electric currents could improve CHX efficacy against multispecies dental biofilms. The bactericidal effect of CHX 0.2% on dental biofilm bacteria was significantly enhanced on Ti surfaces by the addition of 5 mA DCs when used through a customized dental tray. This was not the case when the biofilms had grown on HA discs. Finally, an electricidal effect by itself was also noted on Ti when the biofilms were treated during 5 min with 5 mA DCs and PBS.

Although dental implants brought to dentistry a fantastic implement to improve oral rehabilitations and patients quality of life, some complications notably biological ones have emerged with some concerns since the late 1980s (Mombelli et al. 1987) with the increasing number of placed implants. Peri‐implant diseases, namely peri‐implant mucositis and peri‐implantitis, are now considered to be common complications of oral implants with prevalence estimates of 43 and 22% at the implant level, respectively (Derks and Tomasi 2015). These conditions are assumed to be biofilm‐induced inflammatory diseases that lead to the progressive loss of the surrounding alveolar bone and finally to the implant failure (Lang and Berglundh 2011). Lots of therapeutic approaches (nonsurgical, surgical, with or without antimicrobials) have been proposed to manage these diseases, but currently, none of them have been proven to be highly effective and reliable. There is indeed no evidence suggesting one technique to be better than another to treat peri‐implantitis (Esposito et al. 2012). The initial colonization of oral bacteria and development as a biofilm within the peri‐implant crevice takes place rapidly after implant placement (Quirynen et al. 2006) and can lead in some cases to peri‐implantitis. This inflammatory process is related to the biofilm mode of growth as well as to the nature of the submucosal microflora. Furthermore, the peri‐implant defect morphology as well as the implant macrotopography and microtopography could explain the difficulties to remove efficiently the biofilm and control the disease progression.

Considering these technical and biological issues, new antimicrobial strategies to control dental biofilms on the contaminated implant surfaces are needed to improve the therapeutic outcomes of peri‐implant diseases. One option that has been proposed by the industry is to combine antimicrobial agents with low electric currents in order to enhance their efficacy (Blenkinsopp et al. 1992). This effect was named the bioelectric effect.

The influence of electric currents on biofilm bacteria have been demonstrated in several ways. For instance, some authors showed that electric currents were able to detach 80% of adherent bacteria from a conductive material after a 20‐min treatment time (Hong et al. 2008). It is hence known that surface charge plays an important role in determining the binding force between bacteria and a surface (Song et al. 2015). Generally, bacteria are negatively charged and are more prone to accumulate on positively charged surfaces. Electric currents that generate electric fields could potentially have an impact on the bacterial adhesion, especially on conductive materials. Indeed, they could modify the surface charge properties as to render it repulsive to bacteria (Poortinga et al. 2001). Furthermore, as the extracellular polymeric matrix is negatively charged, electric currents might also lead to biofilm structural changes (expansion/contraction) (Stoodley et al. 1997) that might, as a consequence, interfere with its cohesion and possibly contribute to bacterial detachment.

Additional works have also pointed out that electrical currents had by themselves the ability to kill biofilm and planctonic bacteria (Davis et al. 1989; del Pozo et al. 2009). del Pozo (2009) observed that the killing effect of low DCs (2 mA) on Staphylococcal and Pseudomonas biofilm bacteria could be noted but after several days of action. This antimicrobial effect was called the electricidal effect and was correlated to current intensity and duration. Various hypotheses have been proposed to understand this antimicrobial effect. One could be related to the electrolytic generation of reactive oxygen species (Stewart et al. 1999) or chlorine‐based substances (Davis et al. 1989; Sandvik et al. 2013). Davis et al. (1989, 1991, 1994) showed that electrolytic reactions occurred in media containing chloride ions when submitted to 200–400 μA and led to the production for instance of antimicrobial molecules as free chlorine and chlorine dioxyde. Another interesting hypothesis was recently attributed to the fact that low DCs could promote bacterial autolysis through the increased transcription of positive autolytic regulator genes *in vitro* on a *Staphylococcus aureus* biofilm model (Zhang et al. 2014). This autolysis compromising the cell walls of biofilm bacteria could explain the enhanced effect of gentamicin when supplemented with these microelectric currents. Moreover in 1994, Costerton et al. hypothesized that the electrical enhancement of tobramycin sulfate efficacy they observed against *Pseudomonas aeruginosa* biofilms was likely because of electrophoretic forces that would allow the bactericidal molecules to diffuse more efficiently within the biofilm structure. It is indeed quite well documented now that low electric currents can have by themselves or in conjunction to antimicrobials an effect in removing or killing biofilm bacteria. The mechanisms by which they precisely operate have still to be clarified. Nevertheless, this potency to improve antimicrobial strategies against biofilm bacteria may represent a great interest and relevance to treat refractory periodontitis or peri‐implantitis. The effect of weak electric currents is in accordance with those described previously in the literature on other species or materials. The results were statistically significant on Ti surfaces with an electricidal effect after 5 min using a PBS solution. When 5 mA DCs were supplemented to CHX, they yielded a bioelectric effect that means a synergistic effect. This effect was observed after 30 sec, 1, and 2 min of treatment. Even if this antimicrobial strategy has been a few times documented in industrial or medical research, it is the first time to our knowledge that it is investigated on human multispecies dental biofilms *ex vivo* although previous researches have been performed *in vitro* on single‐ and dual‐species (Lasserre et al. 2015; Wattanakaroon and Stewart 2000) biofilm models. A particular interest for this antimicrobial strategy could emerge for the treatment of peri‐implantitis as no therapeutic approach currently seems to stand out above the others. Furthermore, in the present model, the electrical antimicrobial effect was only observed on titanium and not on HA surfaces. This might be related to the electrical conduction of Ti that is much more important than that of HA. Further studies regarding clinical applications should be conducted to investigate these preliminary promising results.

## Conclusions

The present study shows that new strategies using 5 mA low direct electric currents could be of interest in order to enhance the antimicrobial CHX efficacy against multispecies dental biofilms. An electricidal (with PBS) and a bioelectrical (with CHX) effects were observed *ex vivo* against human dental biofilms on titanium surfaces after 5 min and 30 sec, respectively.

## Conflict of Interest

They declare no conflict of interest.
